# Use of drug-induced sleep endoscopy in Germany—an analysis based on claims data

**DOI:** 10.1007/s11818-023-00398-4

**Published:** 2023-02-18

**Authors:** M. Braun, B. A. Stuck, C. Schöbel, A. Steffen

**Affiliations:** 1 Department of Pneumology, University Medicine Essen—Ruhrlandklinik, West German Lung Center, University Duisburg-Essen, Duisburg, Germany; 2 Faculty of Sleep and Telemedicine, University Medicine Essen—Ruhrlandklinik, West German Lung Center, University Duisburg-Essen, Tueschener Weg 40, 45239 Essen, Germany; 3 Department of Otorhinolaryngology, Head and Neck Surgery, University-Hospital Marburg, Philipps-Universität Marburg, Marburg, Germany; 4 Department of Otorhinolaryngology, University Hospital Schleswig-Holstein Campus Luebeck, University of Luebeck, Luebeck, Germany

**Keywords:** Sleep apnea, obstructive, Nasal surgical procedures, Continuous positive airway pressure, Diagnosis-related groups, Technology adoption, Obstruktive Schlafapnoe, Nasenchirurgische Eingriffe, Kontinuierlicher positiver Atemwegsdruck, Diagnosis Related Groups, Technologienutzung

## Abstract

**Background:**

Drug-induced sleep endoscopy (DISE) has recently gained relevance as a diagnostic tool for obstructive sleep apnea (OSA). However, it is unclear to what extent and in which patient cohorts DISE is used in Germany. With introduction of specific coding for this method in 2021 (*Operationen- und Prozedurenschluessel, *OPS code), usage can now be analyzed based on diagnosis-related groups (DRG) claims data.

**Methods:**

Aggregated data from all inpatient DISE procedures conducted in German hospitals in 2021 were obtained from the publicly available *Institut fuer das Entgeltsystem im Krankenhaus* (InEK) database. Patient-relevant information as well as data on hospitals providing the examinations were exported and analyzed.

**Results:**

Between January and December 2021, a total of 2765 DISE procedures were conducted and documented using the newly introduced specific code (1-611.01). Most patients were male (75.6%), in the age categories 30–39 (15.2%) and 40–49 years (17.2%), and presented with the lowest patient clinical complexity level (PCCL; class 0 = 81.88%). Pediatric use was rare (1.8%). Leading main diagnoses of patients were G47.31 (OSA) and J34.2 (deviation of nasal septum). The most common procedures conducted together with DISE were nasal surgery, and the examination was mostly provided in large public hospitals with more than 800 beds.

**Conclusion:**

Though the OSA prevalence in Germany is high, use of DISE as a diagnostic tool is low and represented only 4.4% of cases with a main diagnosis of OSA in 2021. Since specific coding was only introduced in January 2021, trends cannot yet be identified. Noticeable is the frequent combination of DISE with nasal surgery, which is not obviously related to a diagnosis of OSA. Limitations of the study are mainly related to the underlying data, which are available for the inpatient sector only, and due to potentially limited use of the OPS code, which was introduced recently and might not be known to all hospitals.

## Abstract (de)

**Hintergrund:**

Die medikamenteninduzierte Schlafendoskopie (MISE) hat in den letzten Jahren als Diagnostikverfahren bei obstruktiver Schlafapnoe (OSA) an Bedeutung gewonnen. Unklar ist bislang, wie häufig und bei welchen Patienten die MISE in Deutschland eingesetzt wird. Mit Einführung eines dezidierten Operationen- und Prozedurenschlüssels (OPS) zur Erfassung der Leistungszahlen im stationären und belegärztlichen Sektor ist dies seit 2021 nun möglich.

**Material und Methoden:**

Aus der Datenbank des InEK-Instituts (Institut für das Entgeltsystem im Krankenhaus) wurden alle MISE-Prozeduren identifiziert, die 2021 stationär durchgeführt und mit dem dezidierten OPS kodiert wurden. Informationen zu Haupt- und Nebendiagnosen sowie Merkmalen durchführender Krankenhäuser wurden aus den Abrechnungsdaten exportiert.

**Ergebnisse:**

Zwischen Januar und Dezember 2021 wurden 2765 MISE durchgeführt und über das Diagnosis-Related-Groups (DRG)-System unter Verwendung des spezifischen Prozedurenschlüssels 1‑611.01 kodiert. Die Mehrzahl der untersuchten Patienten war männlich (75,6 %) und wies eine geringe Patientenkomplexität auf („patient clinical complexity level“, PCCL 0 = 81,88 %). Die häufigsten Alterskategorien waren 30–39 Jahre (15,2 %) und 40–49 Jahre (17,2 %). Untersuchungen bei minderjährigen Patienten betrafen 1,9 % der Fälle. Führende Hauptdiagnosen waren G47.31 (OSA) sowie J34.2 (Nasenseptumdeviation), und die häufigsten Prozeduren, die zusätzlich zur MISE durchgeführt wurden, waren Eingriffe am Nasenseptum. Die Mehrzahl der MISE wurde in öffentlichen Kliniken mit mehr als 800 Betten durchgeführt.

**Schlussfolgerung:**

Vor dem Hintergrund der hohen Prävalenz der OSA in Deutschland ist die Nutzung der MISE als diagnostisches Instrument gering und macht nur 4,4 % der gesamten Krankenhausfälle mit dieser Hauptdiagnose im Untersuchungszeitraum aus. Da ein spezifischer Prozedurenschlüssel erst zum Januar 2021 eingeführt wurde, sind Trends noch nicht zu ermitteln. Auffallend ist die regelmäßige Kombination der MISE mit nasenchirurgischen Eingriffen, die nicht offensichtlich in Bezug zur Diagnose OSA stehen. Limitationen der Studie bestehen in der begrenzten Verfügbarkeit der Daten, die sich ausschließlich auf den stationären Sektor beziehen, sowie in einer möglichen Unterdokumentation, da der OPS erst kürzlich eingeführt wurde und nicht allen Kliniken bekannt sein könnte.

Obstructive sleep apnea (OSA) is a well-recognized disorder that can lead to several significant and potentially life-threatening sequelae such as excessive daytime sleepiness, with a higher risk of motor vehicle collisions or cardiovascular diseases [1, 2]. Continuous positive airway pressure and its variants (PAP) are often regarded as first-line treatment for OSA; however, long-term adherence to this treatment is often suboptimal [3]. Therefore, a significant proportion of patients discontinue PAP and remain without sufficient therapy and thus exposed to the risk of OSA-related diseases. Many patients seek alternative treatment, including weight-reduction programs, mandibular advancement devices (MAD), or sleep surgery including hypoglossal nerve stimulation (HNS). For the latter and for pharyngeal surgery, drug-induced sleep endoscopy (DISE) is recognized as a diagnostic tool to identify collapse patterns that are considered as exclusion criteria or risk factors for suboptimal outcomes [4–7]. Consensus recommendations and guidelines for the indication, recommended maneuvers during the examination, and structured evaluation techniques have been established previously [8]. Despite this and its increasing use, DISE was conducted without specific procedural coding in German hospitals until recently.

Based on the International Classification of Procedures in Medicine, which was published by the World Health Organization in 1978, a German modification was introduced in 1994 to allow documentation of diagnostic and therapeutic measures conducted in the German healthcare system (*Operationen- und Prozedurenschluessel*, OPS) [9]. The classification is managed by the German Institute for Medical Documentation and Information (DIMDI), which was merged with the Federal Institute for Drugs and Medical Devices (*Bundesinstitut für Arzneimittel und Medizinprodukte*, BfArM) in 2020 and is updated annually to integrate new methods into the nomenclature. Today, the OPS classification consists of six chapters with more than 230 procedure classes and approximately 19,000 different procedures. This granularity allows procedures to be described in detail, which is required for differentiated reimbursement based on diagnosis-related groups (DRG). In addition, OPS codes are an important source of data for statistical analysis of healthcare services conducted in the German inpatient sector.

For integration of new OPS codes, a specific process was established in which scientific societies, hospitals, federations, and other healthcare institutions can submit an application for modification of an existing code or implementation of a new code [10].

While DISE is used for advanced diagnostics in patients with OSA in many clinics in Germany, a specific OPS code for this procedure was not previously available. To enable specific coding, the working group Sleep Medicine within the German Society of Otorhinolaryngology, Head and Neck Surgery, applied for implementation of a specific code for DISE in January 2020. The application was accepted in July 2020 and a new OPS code “1-611.01 Upper Airway Endoscopy—with flexible endoscope under sedation (sleep endoscopy)” was defined and introduced with the 2021 update of the OPS catalogue [11]. Since DISE is not listed in the German catalogue of outpatient reimbursement (*Einheitlicher Bewertungsmaßstab*, EBM), this examination is only reimbursed by statutory health insurances in hospitals. For patients with private insurance, DISE is usually reimbursed in the outpatient sector as well as in hospitals. To ensure continuous reimbursement and access for all patients, a specific OPS code is thus considered important, as it can also be relevant for further development of DRG-based payments.

## Study objectives

Though DISE is established in the diagnostic management of OSA patients seeking alternative treatment options, no data exist on its actual use in routine clinical practice in Germany. The objective of this study is to analyze the use of this diagnostic tool in the German hospital sector, which is now possible with the introduction of the new OPS code.

## Materials and methods

### Data

Claims data from all patients receiving inpatient diagnostic or therapeutic services in German hospitals can be accessed via the website of the German Institute for the Hospital Remuneration System (*Institut fuer das Entgeltsystem im Krankenhaus*, InEK) [12]. Data are updated three times per year, which allows analysis of the use of healthcare services close to the time of consumption and thus description of a quite recent picture of the actual situation. The dataset obtained for this analysis consisted of demographic information, procedural information, main diagnosis, patient clinical complexity levels (PCCL), and comorbidities, as well as the type of hospital that provided the examination. Data were retrieved for the complete year 2021 on all patients who received a DISE in that period. Additionally, data were obtained for all cases admitted in 2021 with a main diagnosis of OSA, which were identified using the ICD-10 code G47.31 (International Classification of Diseases, German modification, version 10). To account for privacy regulations, aggregated patient data only are available and data are thus reported in categories.

### Statistical analysis

Data were managed using SPSS software (IBM, Armonk, NY, USA, version 26.0). Data items for SARS-CoV 2‑related tests, which were mandatory for hospital admissions during the period evaluated, were excluded from the set, since those were considered nonrelevant for the research question. Standard statistical techniques were used to describe the populations and chi^2^ tests were applied to determine variances between groups.

## Results

In the period from January until December 2021, 2765 procedures were conducted with the DISE-specific OPS code in German hospitals. Most patients were male (75.6%) and in the age groups 30–39 years (15.2%), 40–49 years (17.2%), and 54–59 years (15.2%). The most common diagnoses leading to admission for DISE were obstructive sleep apnea (G47.31, 46.3%) or deviation of the nasal septum (J43.2, 15.1%; Table 1). DISE was often combined with additional procedures during the same hospital stay (Table 2). Of these, nasal surgery (18.1%), other endoscopic examinations, or polysomnography (12.0%) were most often performed during the same admission (Table 2). Implantation of a hypoglossal nerve stimulator (HNS), which requires DISE before treatment to confirm eligibility, was carried out in only 16 patients during the same admission, while 356 cases were performed in 2021. Most DISE examinations for HNS are thus conducted independently of the implantation procedure, during a separate hospital stay or at an outpatient visit. The mean length of hospital stay among all patients receiving DISE was 4.3 ± 10.9 days, while one third (34.9%) had a stay of 1.0 day, which was categorized as a short-term admission.

**Table 1:** Main diagnoses and comorbidities of patients undergoing drug-induced sleep endoscopy (DISE) in Germany (Jan-Dec 2021, DISE admissions: *n* = 2765)

	Description	Cases	% of all cases
**Top 5 main diagnoses in cases where DISE was performed**			
*ICD 10 code*	**–**	**–**	**–**
G47.31	Obstructive sleep apnea	1280	46.3
J34.2	Deviated nasal septum	418	15.1
J34.3	Hypertrophy of nasal turbinates	69	2.5
R06.5	Mouth breathing	52	1.9
J39.2	Other diseases of pharynx	41	1.5
**Top 5 comorbidities in cases where DISE was performed**			
*ICD 10 code*	–	–	–
J34.3	Hypertrophy of nasal turbinates	779	28.2
G47.31	Obstructive sleep apnea	690	24.9
I10.00	Essential (primary) hypertension	671	24.3
J34.2	Deviated nasal septum	471	17.0
R06.5	Mouth breathing	427	15.4

**Table 2:** Other procedures carried out in patients admitted to hospital for drug-induced sleep endoscopy (DISE) in Germany (Jan-Dec 2021, DISE admissions: *n* = 2765)

OPS code	Description	Cases	% of all cases
1‑610.0	Diagnostic laryngoscopy	513	18.6
5‑215.4	Surgery on inferior turbinates: lateralization	500	18.1
1‑611.00	Diagnostic pharyngoscopy, direct	473	17.1
5‑214.6	Submucosal resection and plastic reconstruction of the nasal septum: plastic correction with resection	465	16.8
5‑215.00	Surgery on inferior turbinates: destruction with diathermy	463	16.7
1‑630.1	Diagnostic esophagoscopy	376	13.6
1‑790	Polysomnography	332	12.0
8‑930	Monitoring of respiration, heart, and circulation without measuring pulmonary artery pressure and central venous pressure	308	11.1
1‑612	Diagnostic rhinoscopy	301	10.9
1‑611.0x	Diagnostic pharyngoscopy, direct, other techniques	299	10.8

Patients receiving DISE often presented with comorbidities (Table 1). Commonly found were diseases of the nose and paranasal sinuses (52.9%), arterial hypertension (29.4%), obesity (14.4%), or diabetes type II (6.6%).

### Comparison to patients hospitalized for OSA

During the same time period as included in the analysis, a total of 60,451 patients were admitted to German hospitals for OSA-related diagnostic or therapeutic measures (Table 3). The length of stay in these patients was shorter than for DISE admissions, with a mean of 1.8 ± 1.5 days and 37.7% short-term admissions.

**Table 3:** Diagnostic procedures carried out in patients admitted to hospital with main diagnosis of obstructive sleep apnea (OSA; G47.31) in Germany (Jan-Dec 2021, OSA admissions: *n* = 60,451)

OPS code	Description	Cases	% of all cases
1‑790	Polysomnography	50,397	83.2
1‑710	Plethysmography (whole body)	9366	15.5
1‑711	Measurement of CO lung diffusion capacity	5000	8.3
1‑791	Cardiorespiratory polygraphy	3366	5.6
1‑245	Rhinomanometry	1830	3.0
1‑795	Multiple sleep latency test (MSLT)/maintenance of wakefulness test (MWT)	1378	2.3
1‑713	Measurement of functional residual capacity (FRC)	1359	2.2
1‑611.01	Drug-induced sleep endoscopy^a^	1280	2.1
1‑900.0	Psychosomatic and psychotherapeutic diagnostic	686	1.1
1‑715	Six-minute walk test	603	1.0

^a^Missing cases to total DISE admissions were coded with different main diagnoses (see Table 1)

**Fig. 1:**
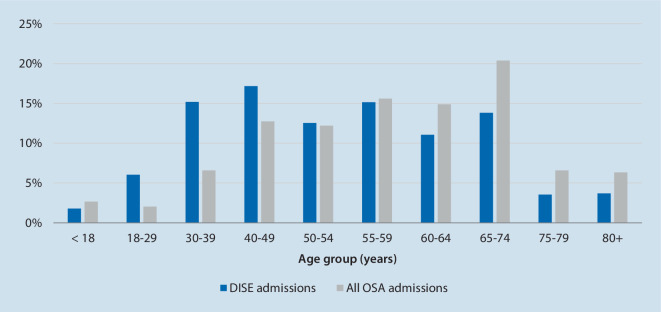
Age distribution of patients receiving drug-induced sleep endoscopy (*DISE*) compared to all obstructive sleep apnea (*OSA*) admissions in German hospitals in 2021 (DISE admissions: *n* = 2765)

**Fig. 2:**
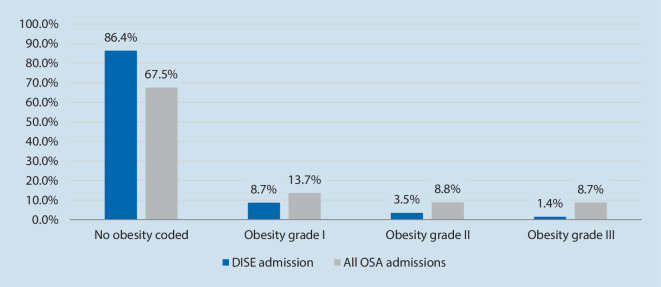
Obesity grade in patients undergoing drug-induced sleep endoscopy (*DISE*) in German hospitals vs. all patients admitted for obstructive sleep apnea (*OSA*; using World Health Organization obesity classification; missing to 100% = obesity, not classified; DISE admissions: *n* = 2765)

The cohort of patients admitted for DISE differed in various aspects to those hospitalized for OSA in general. As such, patients with OSA in general had a different age distribution (Fig. 1) and were more often of female gender (30.3 vs. 24.4%, *p* < 0.001). Additionally, patients hospitalized for OSA in general were significantly less healthy, which is represented by higher PCCL scores (Table 4). Patients undergoing DISE presented significantly less often with comorbid conditions such as arterial hypertension (29.4 vs. 37.2%, *p* < 0.001), coronary artery disease (3.4 vs. 5.6%, *p* < 0.001), congestive heart failure (1.5 vs. 2.3%, *p* *=* 0.003), or type II diabetes (6.6 vs. 11.2%, *p* < 0.001) than patients admitted for OSA in general. Obesity was significantly more prevalent in the general group of OSA patients (14.4 vs. 32.5%, *p* < 0.001). Among those with OSA and obesity, 17.5% had a body mass index (BMI) of > 35 kg/m^2^ and 8.4% were highly obese, with a BMI > 40 kg/m^2^, compared to 4.9 and 1.4% of cases, respectively, in the DISE cohort (Fig. 2).

**Table 4:** Characteristics of patients admitted for DISE compared to all patients admitted with a main diagnosis of OSA

	DISE admissions (%)	All OSA admissions (%)	*p*-value
Gender (m/w, %)	75.6/24.4	69.7/30.3	< 0.001
Obesity (WHO grade ≥ 1)	14.4	32.5	< 0.001
Arterial hypertension	29.4	37.2	< 0.001
Coronary heart disease	3.4	5.6	< 0.001
Congestive heart failure	1.5	2.3	0.003
Diabetes mellitus type II	6.6	11.3	< 0.001
*Patient comorbidity and complications level*			
PCCL class 0	85.6	86.7	0.088
PCCL class 1	4.8	5.5	0.118
PCCL class 2	3.6	5.6	< 0.001
PCCL class 3	3.1	2.0	< 0.001
PCCL class 4	1.9	0.1	< 0.001
PCCL class 5	0.8	0.1	< 0.001
PCCL class 6	0.1	0.0	< 0.001

*DISE* drug-induced sleep endoscopy, *OSA* obstructive sleep apnea, *WHO* World Health Organization, *PCCL* patient clinical complexity level

**Fig. 3:**
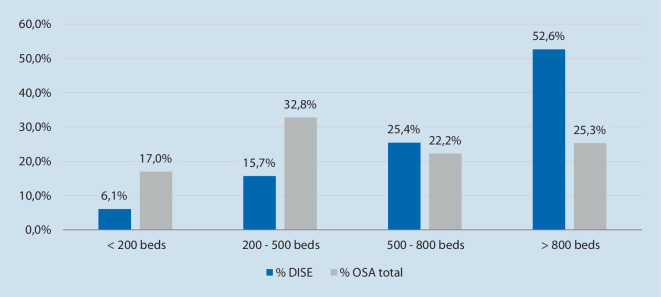
Distribution of drug-induced sleep endoscopy (*DISE*) and total obstructive sleep apnea (*OSA*) cases across German hospitals in 2021 by hospital size (DISE admissions: *n* = 2765; OSA admissions: 60,451)

While in theory, DISE can be conducted at any hospital in Germany with a specialist trained in this technique, the examination is disproportionately provided across hospitals (Fig. 3). While large institutions with more than 800 beds admitted only 25.3% of all OSA patients, they conducted 52.6% of all DISE procedures. On the other hand, hospitals with less than 200 beds conducted only 6.1% of all DISE procedures in the period analyzed, while they provided care for 19.7% of all OSA cases.

## Discussion

The clinical value of DISE has been proven in multiple trials and it is recommended as a diagnostic tool in patients with OSA seeking PAP alternatives, especially in screening for MAD and HNS or for planning of surgical interventions on the upper airway [13, 14]. As expected, the examination is used in Germany, but in the absence of a specific procedure code, it was not possible to estimate the actual use until now. This study provides a first analysis of routine clinical practice in German hospitals, and presents some interesting findings.

Although DISE was introduced many years ago, its relevance as a diagnostic tool for patients with OSA in Germany is limited. In 2021, only 2765 examinations were conducted in the hospital sector, which represents 4.5% of all admissions for OSA. Other countries that use DISE in routine clinical practice report remarkably higher use. A recent analysis from the Netherlands, for example, stated 7090 examinations in 2018, while this country has only a fifth of the German population [15].

With most cases performed by hospitals with more than 800 beds, DISE is a method that is largely provided by clinics with a higher level of specialization. Though no data are available on the medical discipline that conducts the examination, it is likely that these will be mainly larger otorhinolaryngology or pneumological departments that specialize in advanced diagnosis and treatment of OSA. As interpretation of DISE findings and subsequent therapeutic decision-making requires training and experience, it seems reasonable that this examination is mainly provided by specialized centers with higher caseloads.

Patients receiving DISE are often admitted for a combination of examinations or interventions. Interestingly, 15% of patients receiving DISE were admitted with a main diagnosis of nasal septum deviation and 18% received DISE in combination with some type of nasal surgery. Though it does not seem logical to admit a patient with such a diagnosis for DISE, which is not obviously linked to OSA, there are a few reasons that could explain the high number of patients with nasal main diagnoses undergoing DISE. First, snoring is one of the leading symptoms in OSA and patients might be admitted with this diagnosis before OSA is confirmed, i.e., in a hospital stay that combines polysomnography and DISE. Secondly, as DISE is conducted largely by otorhinolaryngologists, concurrent septal deviation or other nasal conditions that require treatment are performed in the same hospital stay and under the same general anesthesia as the DISE to reduce the burden for the patient and streamline the treatment process. Conducting DISE at the same time as other surgeries also explains the relatively long mean hospital stay of 4.3 days, which would not be required for DISE alone. With its low invasiveness, the examination is commonly carried out as an outpatient procedure in other healthcare systems [8]. Due to limitations to reimbursement within the German statutory health insurance system, this is possible only in private or self-paying patients, and those cases are not documented in any statistic. With introduction of the specific OPS code, reimbursement of DISE is now possible in hospitals via DRG payments. Given constant budget constraints, it remains to be seen whether the new code will lead to improved funding of this examination technique. Based on anecdotal feedback from some clinics providing DISE, payers often deny claims ex-post, even if proper coding was used and the procedure was conducted per standards of care.

The population receiving DISE consists largely of male patients of the typical age groups in which symptoms become apparent. Although patients present with the typical comorbidities, they tend to be rather healthy, with only few classified as more complex in the respective higher PCCL groups. As such, the population receiving DISE can be considered healthier when compared to the general population admitted for OSA. This is supported by the significantly higher rates of all comorbidities investigated in the population of all admitted OSA patients.

Of particular interest is the distribution of obesity levels across the two groups: patients with higher levels of obesity are underrepresented among patients receiving DISE. Given that accumulation of fat tissue in the upper airway can lead to more complex anatomical situations, it is surprising that DISE is not used more often in this population, since it could lead to improved understanding of the pathophysiology and thus better decision-making. Specific reasons cannot be derived from the available data, as the particular indication for the examination and the phase of the patient in their disease journey is not documented. Potentially, DISE is mainly used in patients who undergo screening for treatments that have specific BMI limits, such as MAD or HNS therapy. Another explanation could be that DISE is mainly used in cases with low adherence to PAP therapy to evaluate potential second-line treatments, which is more common in patients with lower levels of obesity [16]. Additionally, use of DISE will certainly be influenced by individual patient preferences, daytime symptoms, and treatment motivation. Given the fact that this examination is mainly provided by larger hospitals, accessibility across the country will likely also drive demand from patients.

### Limitations

This study is subject to certain limitations, which are mainly related to the dataset that was available for analysis. First, the specific OPS code was only implemented in the analyzed year. Some providers might not have been aware that DISE can be documented with this code and some cases could have been performed using the nonspecific coding that was available beforehand. Second, the study only includes DISE cases performed during a hospital admission that led to billing of a DRG. Hospitals with specific agreements for ambulatory care or which use other reimbursement schemes could not be obliged to use the new code. Since DISE is also offered by physicians in the outpatient sector for privately insured or self-paying patients, these cases are also not included, as there is no requirement to report them. As such, there could be an underreporting of the actual DISE cases performed in 2021. As reimbursement of DISE by statutory insurance is only available for hospitals and these payers covers 88% of the German population, we believe, however, that the dataset covers the vast majority of examinations [17]. Another limitation is the completeness of the dataset itself, which provides information at only an aggregated level and in limited granularity; therefore, a more advanced analysis including patient pathways and disease journeys was not possible. This would, however, be interesting to further evaluate this technique for diagnosis of patients with OSA.

## Conclusion

With introduction of specific coding for DISE in Germany, it is now possible to analyze its use based on DRG claims data. This article provides first insights into the role of this examination technique as a diagnostic tool in OSA patients and the cohort receiving DISE in German hospitals. The relevance of this tool in the diagnostic workup of these patients is relatively low, however, compared to the prevalence of the disease. Although this is only a first analysis, it can be concluded that patients receiving DISE are younger, less obese, and present with fewer comorbid conditions. Given that PAP alternatives are not only required in this cohort, these findings may be relevant, since they might lead to improved access to DISE, so that more patients can receive this examination in a work-up for advanced diagnosis of OSA and potential second-line treatments.

## Declarations

Ethics committee approval was not required for this study. For this article, no studies with human participants or animals were performed by any of the authors. All studies mentioned were in accordance with the ethical standards indicated in each case.

## References

[1] Benjafield AV (2019). Estimation of the global prevalence and burden of obstructive sleep apnoea: a literature-based analysis. Lancet Respir Med.

[2] Stuck BA (2020). Teil-Aktualisierung S3-Leitlinie Schlafbezogene Atmungsstörungen bei Erwachsenen.

[3] Fietze I (2020). Wenn CPAP nicht genutzt oder nicht vertragen wird – Vorschlag für eine standardisierte Terminologie. Somnologie.

[4] Van de Heyning PH (2012). Implanted upper airway stimulation device for obstructive sleep apnea. Laryngoscope.

[5] Hasselbacher K, Bruchhage K-L, Abrams N, Steffen A (2018). Sleep endoscopy and complete concentric collapse in CPAP failure. HNO.

[6] Green KK (2019). Drug-induced sleep endoscopy and surgical outcomes: a multicenter cohort study. Laryngoscope.

[7] Huyett P (2021). Drug-induced sleep endoscopy and Hypoglossal nerve stimulation outcomes: a multicenter cohort study. Laryngoscope.

[8] De Vito A (2018). European position paper on drug-induced sleep endoscopy: 2017 Update. Clin Otolaryngol.

[9] Bundesinstitut für Arzneimittel und Medizinprodukte (BfArM, “Operationen- und Prozedurenschlüssel (OPS).”). https://www.bfarm.de/DE/Kodiersysteme/Klassifikationen/OPS-ICHI/OPS/_node.html (Created 1 Jan 2022). Accessed 26 July 2022

[10] BfArM – Vorschlagsverfahren. https://www.bfarm.de/DE/Kodiersysteme/Klassifikationen/Vorschlagsverfahren/_node.html. Accessed 26 July 2022

[11] Steffen A “OPS-Änderungsvorschlag 221-2021: Differenzierung: Flexible transnasale Pharyngoskopie im Liegen als Schlafendoskopie.” Bundesinstitut für Arzneimittel und Medizinprodukte (BfArM). https://www.bfarm.de/DE/Kodiersysteme/Services/Downloads/OPS/_functions/ops-vorschlaege-2021.html?nn=841246&cms_gtp=1004626_list%253D9 Accessed 26 July 2022

[12] Institut für das Entgeltsystem im Krankenhaus GmbH (InEK GmbH) InEK Datenbrowser. https://datenbrowser.inek.org/ Accessed 26 July 2022

[13] Mayer G (2017). German S3 Guideline Nonrestorative Sleep/Sleep Disorders, chapter ‘Sleep-Related Breathing Disorders in Adults’, short version: German Sleep Society (Deutsche Gesellschaft für Schlafforschung und Schlafmedizin, DGSM). Somnologie.

[14] Van den Bossche K (2022). Multimodal phenotypic labelling using drug-induced sleep endoscopy, awake nasendoscopy and computational fluid dynamics for the prediction of mandibular advancement device treatment outcome: a prospective study. J Sleep Res.

[15] Zinnige Zorg: Verbetersignalement Obstructieve slaapapneu. https://www.zorginstituutnederland.nl/publicaties/rapport/2021/03/05/zinnige-zorg---verbetersignalement-obstructieve-slaapapneu (Created 23 Feb 2021). Accessed 26 July 2022

[16] Gray EL, McKenzie DK, Eckert DJ (2017). Obstructive sleep apnea without obesity is common and difficult to treat: evidence for a distinct pathophysiological phenotype. J Clin Sleep Med.

[17] Verband der Ersatzkassen Daten zum Gesundheitswesen: Versicherte. https://www.vdek.com/presse/daten/b_versicherte.html (Created 25 Feb 2022). Accessed 8 Aug 2022

